# Guillain-Barre Syndrome Amid Osteosarcoma Treatment: A Therapeutic Dilemma and Literature Review

**DOI:** 10.7759/cureus.12432

**Published:** 2021-01-02

**Authors:** Geraldine Peralta Suarez, David W Deng, Raquel Silva, Gabriel Tinoco

**Affiliations:** 1 Internal Medicine, Universidad Militar Nueva Granada, Bogota, COL; 2 Department of Internal Medicine, The Ohio State University College of Medicine, Columbus, USA; 3 Physical Medicine and Rehabilitation, OhioHealth, Columbus, USA

**Keywords:** guillain-barre syndrome (gbs), osteosarcoma, adjuvant chemotherapy, sarcoma

## Abstract

Guillain-Barre syndrome (GBS) is a clinical syndrome with multiple variants. GBS is defined as an acute demyelinating polyneuropathy commonly preceded by infection (bacterial or viral), trauma, or inflammatory processes, which triggers an autoimmune response that affects the peripheral nervous system. This case report describes a patient with high-grade osteosarcoma that completed neoadjuvant chemotherapy and underwent surgical resection with no immediate complications. Fourteen days after the surgery, the patient developed an acute inflammatory demyelinating polyradiculopathy consistent with GBS. As the five-year survival without chemotherapy is only around 20%, this challenging clinical scenario raised questions regarding adjuvant chemotherapy's safe completion in this setting.

## Introduction

Guillain-Barre syndrome (GBS) is defined as an acute demyelinating polyneuropathy, which is currently best understood as a monophasic autoimmune condition affecting the peripheral nerves [1]. It is thought that GBS's pathophysiology is related to both cell-mediated and humoral immunity [2]. The incidence in Europe and North America is roughly 0.16-4/100.000 habitants per year and 0.5-1.5/100.000 habitants per year in adults and children, respectively. The highest rates are seen in the elderly (>80 years) and are usually 1.5 times more common in males than females. Frequently patients describe symptoms like fever (52%), cough (48%), sore throat (39%), nasal discharge (30%), and diarrhea 2-4 weeks before the onset of GBS [1, 2].

The clinical spectrum of Guillain-Barré syndrome is wide-ranged. Multiple variants have been identified: acute inflammatory demyelinating polyneuropathy (AIDP) and axonal subtypes - acute motor axonal neuropathy (AMAN) and acute motor and sensory axonal neuropathy (AMSAN). The clinical features of those types of GBS are similar. However, the course is faster and more severe in the axonal form, which also more frequently causes both respiratory and cranial nerve involvement and less frequently causes dysautonomia [2].

Bacterial infections are most commonly associated with GBS, most notably Campylobacter jejuni (C. jejuni). The pathophysiology of prior C. jejuni infection-causing GBS is explained in the literature by molecular mimicry between C. jejuni lipooligosaccharide and gangliosides (which are abundant in nervous tissue) [1, 2]. Certain other conditions have been proposed as possibly predisposing patients to GBS, including vaccination, surgery, trauma, bone marrow transplant, cancer, and chemotherapy [2].

Cases of GBS have been reported after administration of a single vaccine or combination of two vaccines. The highest rate of cases was observed after the swine influenza vaccination in 1976-1977. Five hundred thirty-two patients received influenza vaccination in New Jersey before the onset of the Guillain-Barre symptoms. This vaccine contained contaminating residues that triggered an autoantibody response against GM1 in the host. The mean interval from vaccination to onset of symptoms was 3.9 weeks, and the estimated risk of vaccine-related GBS in adults was roughly one case per 100,000 vaccinations. The second most frequently reported cause post-vaccination GBS was hepatitis vaccination (nine out of 54 patients). The association with tetanus and diphtheria toxoid vaccine was found in a lower proportion (one out of 54 patients). However, current literature suggests that the presentation of GBS after vaccination is extremely rare [3].

Mayo Clinic conducted a trial in which 208 patients were diagnosed with GBS within six weeks after surgery. It was found that comorbid autoimmune disease was associated with the development of post-surgical GBS. Cancer patients were more susceptible to develop GBS, with a reported prevalence of 2.4 times higher than the general population. It has been hypothesized that surgery produces transient immunosuppression that results in autoantibodies' generation to promote an attack on peripheral nerves; however, its physiopathology remains poorly understood [4, 5]. The literature also describes that patients after trauma can develop GBS, specifically the axonal subtype, more commonly than the demyelinating subtype. It is essential to differentiate it from critical illness polyneuropathy, which is common after trauma [1, 2].

The link between GBS and chemotherapy has not been established yet; however, a possible explanation for this association is that the treatment generates a transient state of immunosuppression that can increase immunologically mediated damage to the myelin sheath catalyzed by the tumor epitope [6]. Also, platinum chemotherapy could increase the production of circulating TNF-a and interleukin-6 involved in the development of GBS [7].

On the other hand, GBS associated with solid tumors and hematological malignancies is rare; however, GBS cases associated with lymphomas, leukemias, lung, breast, and colon cancer have been reported [7]. Tumor cells can produce onconeural antigens that stimulate onconeural antibody production like molecular mimicry in infectious processes [6]. Hocker et al. showed that post-surgical GBS had a higher incidence in patients with an active malignancy than those who did not have cancer [4]. The treatment of GBS generally includes IVIG, plasmapheresis, and supportive care. This entity's clinical course has a high degree of variability, and occasionally patients clinically worsen after showing initial signs of clinical improvement.

High-grade osteosarcoma is the most common primary bone malignancy, which also infrequently affects soft tissues. It is generally a disease that affects children and young adults or older individuals with a bimodal age distribution. Historical data shows a survival rate of around 20% with surgery alone in the pre-chemotherapy era. The modern standard treatment of localized osteosarcoma includes perioperative chemotherapy and surgery with a five-year survival exceeding 70% and long-term survival benefits. Although the optimal chemotherapy regimen has not been fully established, data available shows it is of paramount importance to complete the adjuvant chemotherapy [8, 9].

## Case presentation

A 55-year-old man was diagnosed with localized (stage IIb) high-grade osteosarcoma of the left humerus. He completed his neoadjuvant chemotherapy with two cisplatin cycles (60mg/m2 x 2 days) and doxorubicin (37.5 mg/m2 x 2 days) with excellent tolerance and minimal side effects. The patient underwent resection of the primary tumor and total humeral replacement 43 days after receiving the chemotherapy, with no complications. Notably, his pathology report showed excellent chemotherapy response with over 95% tumor necrosis and negative margins.

Fourteen days after the surgery, he presented to the emergency department with severe lower back pain and bilateral weakness and numbness and tingling in his anterior thighs. There was no recent history of trauma, infection, ill contacts, or vaccination. Initially, computed tomography of the spine showed no acute fractures/misalignment and no suspicious osseous lesions. The magnetic resonance of the spine showed no signs of cord compression or metastatic disease. On exam, he had 4/5 weakness in his hip flexions, knee extensors and flexors, areflexia through his toes, and no sensory loss. Further workup with lumbar puncture revealed protein elevated at 88 mg/dl (reference: 15-45 mg/dL) and normal white blood cells at 3 /uL (reference: <6 /uL). The differential diagnosis included chemotherapy-related neuropathy, post-surgical mono neuritis multiplex, and infection. Extensive workup ruled out infectious processes, and given the clinical presentation, GBS or acute inflammatory demyelinating polyradiculopathy (AIDP) was deemed most consistent with the patient's symptoms.

The patient received intravenous immunoglobulin (2 mg/kg day) for a planned five days. Unfortunately, the patient's neurological symptoms worsened initially, as he developed right facial paresthesia and droop that progressed to bilateral facial weakness. His hip flexion and knee extension worsened to 2/5 strength, he developed urinary retention and had dysmetria in his right upper extremity. Electromyography and nerve conduction studies were consistent with sensorimotor polyneuropathy with demyelinating features. Fifteen days after his initial presentation, the patient had some neurological improvement, 5/5 muscle strength in the right arm, 3/5 strength in the lower extremities, and improved facial weakness. Shortly after that, the patient was discharged from the hospital and received an extensive inpatient rehabilitation program, with excellent response.

Clinically his neurological symptoms stabilized, and due to the concern for worsening neuropathy with the continued cisplatin use, it was decided to complete his adjuvant chemotherapy with single-agent doxorubicin. The chemotherapy was started 12 weeks after the initial presentation in the emergency department. He completed his adjuvant chemotherapy with minimal side effects. He continued his rehabilitation and was able to regain the ability of daily living activities independently, and to date, he has successfully participated in four half marathons. Thirty months after his surgery, there is no evidence of recurrence from osteosarcoma or GBS.

## Discussion

Our patient met the Brighton criteria for Guillai- Barre's diagnosis (level 1 of diagnostic certainty). To our knowledge, this is the first case reported of GBS occurring in an adult patient receiving curative treatment for high-grade osteosarcoma. It is not clear the exact trigger of GBS; however, associated factors include chemotherapy, surgery, and the malignancy itself [1, 2, 4].

Cisplatin is notorious for causing neuropathy as one of its adverse effects [10]. This neuropathy is described as an axonal, mostly sensory polyneuropathy, classically involving a "stocking-glove distribution'" which is different and much more benign than the more typical presentation of GBS. Yet, it should be noticed GBS infrequently can present as ascending paralysis in a "stocking-glove distribution" [11]. There are case reports of GBS occurring after treatment with platinum-based chemotherapy in patients with solid organ cancers, notably endometrium, lung, and colon cancer [6, 12, 13]. Reported cases of chemotherapy-associated GBS are presented in Table 1.

**Table 1:** Reported cases of chemotherapy-associated Guillain-Barre syndrome

Chemotherapy regimen	Cumulative dose	Onset of symptoms	Signs and symptoms
Cisplatin [6]	240 mg/m2	30 days	Lower limb weakness, mild distal upper limb weakness, ankle and knee reflexes absent, sensory impairment in soles
Cisplatin and gemcitabine [13]	144 mg and 1800 mg	Six days	Tetraparesis, lower limb areflexia, facial palsy
Cisplatin, etoposide, and bleomycin [7]	300 mg/m2, 1500 mg/m2, and 270 mg	Seven days	Tetraparesis, hypoesthesia, generalized areflexia, dyspnea, micturition impairment
Bevacizumab [14]	Unclear	30 days	Tetraparesis, dyspnea, generalized areflexia, swallowing disturbance
Pazopanib [14]	800 mg/day for 34 days	30 days	Symmetrical distal limb weakness, sensory disturbance, areflexia
Cisplatin, doxorubicin, and cyclophosphamide (CAP) [15]	Unclear	After six cycles of CAP	Ascending paralysis, absence of deep tendon reflexes.

Pappa et al. directed a study of 449 patients diagnosed with peripheral neuropathies between 2012 and 2019. Five patients met the Brighton criteria for GBS diagnosis, and all patients had received platinum for the treatment of their tumors. They found that in all five cases, the cumulative platinum doses were below the established threshold for neurological toxicity (less than 300-450 mg/m2 of cisplatin). Patients presented marked motor compromise and disability for walking at the peak of disease severity. This compromise is consistent with GBS's clinical features vs. toxic peripheral neuropathy, characterized by sensory ataxia and distal painful paresthesias [16].

There is also a case report of GBS occurring after doxorubicin-based chemotherapy. With our patient's GBS 47 days after receiving doxorubicin and cisplatin, it does not exactly fit in with the case reports that cite GBS occurring after cisplatin chemotherapy (reported as happening as 1-2 weeks after chemotherapy). However, the case report by Terenghi et al. [17] does report initial symptoms presenting six weeks after treatment containing doxorubicin. To date, the use of single-agent Adriamycin has not been linked with GBS.

In another case of GBS after treatment with doxorubicin, bleomycin, vinblastine, and dacarbazine (ABVD), it was decided to hold vinblastine for one cycle and reduce the dose in another before reintroducing it all the full dose in the last cycle. It remains unclear the optimal chemotherapy regimen for adult patients with localized high-grade osteosarcoma. Still, in general, high-dose methotrexate (younger adults), doxorubicin, cisplatin, and ifosfamide are used [8].

The completion of adjuvant chemotherapy is paramount for treating high-grade osteosarcoma, as the five-year survival without chemotherapy is only around 20% [8, 9]. This challenging case raises a clinical dilemma: could adjuvant chemotherapy be safely completed despite a very recent occurrence of GBS? Or avoiding further chemotherapy and therefore reducing the osteosarcoma cure rate to avoid possible GBS re-occurrence is warranted? 

After an exhaustive review of the literature, and extensive discussion with the patient and his family as well as our multidisciplinary sarcoma team, it was decided to discontinue cisplatin to avoid worsening neuropathy, and we decided to proceed with adjuvant single-agent doxorubicin, as historically anthracyclines constitute the backbone of the high-grade osteosarcoma [8]. To date, no studies have been published for patients with high-grade osteosarcoma, demonstrating alternative adjuvant treatments with similar efficacy compared to the standard of care nor the outcomes of patients that did not receive adjuvant cisplatin.

One study describes an increase in cancer incidence in patients with GBS vs. patients without GBS, suggesting a possible association [19]. The study also evidenced the possibility of GBS as a paraneoplastic syndrome; however, this has never been reported in osteosarcoma patients [20]. Our patient did not show any evidence of paraneoplastic syndrome.

It has been hypothesized that tumors could produce antigens that cause autoimmunity against the peripheral nerves; this is seen in notable paraneoplastic syndromes that affect the nervous system [15]. Another hypothesis suggests that the relative immunosuppression related to cancer or its treatment could lead to GBS through immune regulation defects [15]. The underpinnings of the possible immune-mediated mechanisms remain unclear at the present time.

The medical literature also reports an increased incidence of GBS in patients after surgery than patients not undergoing surgery [1, 2, 4, 5]. However, the incidence is exceedingly low compared to the incidence of GBS after well-known infectious triggers. It has been hypothesized that transient relative immunosuppression from the release of endogenous glucocorticoids after surgery may explain this increased incidence [2]. In our patient, the onset of symptoms occurred only two weeks after surgery, making surgery highly unlikely as the triggering event for GBS.

## Conclusions

GBS is a rare and often devastating entity, with unclear physiopathology, variable presentation, and limited treatment options. To our knowledge, this is the first case reported of GBS occurring in an adult patient receiving curative treatment for high-grade osteosarcoma. Anthracycline-based chemotherapies could be administered with caution once GBS is in the recovery phase. The risks and benefits of the therapy should be thoroughly discussed with the patient and a multidisciplinary team.
